# Indigenously produced biochar retains fertility in sandy soil through unique microbial diversity sustenance: a step toward the circular economy

**DOI:** 10.3389/fmicb.2023.1158784

**Published:** 2023-06-27

**Authors:** Munawwar Ali Khan, Alsayeda Zahra Salman, Shams Tabrez Khan

**Affiliations:** ^1^Department of Life and Environmental Sciences, College of Natural and Health Sciences, Zayed University, Dubai, United Arab Emirates; ^2^Department of Systems Biology, College of Life Science and Biotechnology, Yonsei University, Seoul, Republic of Korea; ^3^Department of Agricultural Microbiology, Aligarh Muslim University, Aligarh, Uttar Pradesh, India

**Keywords:** microbial community, biochar, arid soil, salinity stress, plant growth promotion

## Abstract

**Introduction:**

Agricultural productivity in the arid hot desert climate of UAE is limited by the unavailability of water, high temperature, and salt stresses. Growing enough food under abiotic stresses and decreasing reliance on imports in an era of global warming are a challenge. Biochar with high water and nutrient retention capacity and acid neutralization activity is an attractive soil conditioner. This study investigates the microbial community in the arid soil of Dubai under shade house conditions irrigated with saline water and the shift in the microbial community, following 1 year of amendment with indigenously prepared biochar from date palm waste.

**Methods:**

Amplicon sequencing was used to elucidate changes in bacterial, archaeal, and fungal community structures in response to long-term biochar amendment. Samples were collected from quinoa fields receiving standard NPK doses and from fields receiving 20 and 30 tons ha^−1^ of biochar, in addition to NPK for 1 year. Water holding capacity, pH, electrical conductivity, calcium, magnesium, chloride, potassium, sodium, phosphorus, total carbon, organic matter, and total nitrogen in the soil from biochar-treated and untreated controls were determined.

**Results and discussion:**

The results show that soil amendment with biochar helps retain archaeal and bacterial diversity. Analysis of differentially abundant bacterial and fungal genera indicates enrichment of plant growth-promoting microorganisms. Interestingly, many of the abundant genera are known to tolerate salt stress, and some observed genera were of marine origin. Biochar application improved the mineral status and organic matter content of the soil. Various physicochemical properties of soil receiving 30 tons ha^−1^ of biochar improved significantly over the control soil. This study strongly suggests that biochar helps retain soil fertility through the enrichment of plant growth-promoting microorganisms.

## 1. Introduction

The sharp rise in human population is coupled with increased food demand and surging anthropogenic activities. The agriculture sector is facing the challenge of meeting this demand in a changed climate, triggered by global warming and shrinking area under cultivation. As the major soil type in UAE is Typic Torripsamments having an aridic moisture regime (Raj et al., 2022), agriculture in this sandy soil is interrupted by long droughts, and salinity is threatening the food security of the country. However, UAE has prioritized self-sufficiency through sustainable food production in accordance with the United Nations Millennium Development Goal to eradicate extreme poverty and hunger (Food and Agriculture Organization of the United Nations, 2017). Sandy soil is nutrient poor and has low nutrient and water retention capacities, requiring frequent irrigation (Yost and Hartemink, 2019). Scarce rainfall, high temperatures, and changing climate advocate the need to conserve soil properties and water and prevent nutrient depletion. Development of stress-tolerant plant varieties, soil amendment with organic and inorganic matter, and use of consortia-based biofertilizers for sustaining soil fertility are some of the focus areas to achieve the goal (Chen et al., 2011; Khan and Khan, 2020; Khan and Malik, 2021; O'Brien et al., 2021; Khan, 2022).

Biochar obtained by pyrolyzing solid biomass such as animal manure, sewage sludge, algae, crop residue, and wood chips under oxygen-free conditions is an attractive soil conditioner for sustaining and improving soil fertility (Ding et al., 2016; Głab et al., 2016; Mansoor et al., 2021). It also helps in enhancing soil carbon content which remains stable, resists degradation, and persists in soil for years (Woolf et al., 2010; Lehmann et al., 2011). Biochar can also enhance soil's physical and chemical properties, such as soil structure, bulk density, porosity, pH, and electrical conductivity (Kumar and Bhattacharya, 2023). It improves water retention capacity and nutritional status of the soil, consequently improving crop yield (Ding et al., 2016; Głab et al., 2016; Mansoor et al., 2021). Microbes play a major role in improving soil fertility through residue decomposition and increasing nutrient availability for plant uptake (Suzuki et al., 2005; Khan, 2022). Biochar in the soil can act as a niche for microbial habitation. It provides nutrients to microbes supporting their enrichment and consequently improving microbe-mediated soil biogeochemical processes. Most of the studies on biochar are short-termed and report the response of growth-promoting rhizobia to biochar amendment (Çig et al., 2021).

Experiments evaluating the influence of biochar amendments and irrigation with saline water on the growth of quinoa plants in the UAE have been reported recently (Gill et al., 2020; Rezzouk et al., 2020). However, studies defining indigenous microbial populations in this arid soil ecosystem are not available. How does the soil microbial community respond to a yearlong amendment with biochar? How the physicochemical properties of soil are influenced by the 1-year long addition of biochar? Finally, whether these changes can be correlated with the changes in microbial diversity. This study addresses these questions intending to improve agricultural productivity in this region.

## 2. Materials and methods

### 2.1. Biochar preparation and study design

The experiments reported in this study are part of studies conducted at the International Center for Biosaline Agriculture (ICBA), UAE, to evaluate the change in the biomass yield of quinoa (*Chenopodium quinoa*), following the amendment of agricultural soil with various doses of biochar. The focus of the study reported here, however, was to assess the impact of biochar addition on soil fertility with special reference to changes in microbial community structure. The change in soil physicochemical properties in response to biochar addition was also checked to establish whether these changes have any significant impact on microbial community structure. The biochar used in this study was produced at ICBA through pyrolysis of date palm residues, mainly dried date palm leaves at 350°C. Before the final application to the field, the biochar was crushed and passed through a 2 mm sieve (Gill et al., 2020). The biochar produced was subjected to a quality control check, and its characteristics are described elsewhere (Gill et al., 2020).

**Figure 1:**
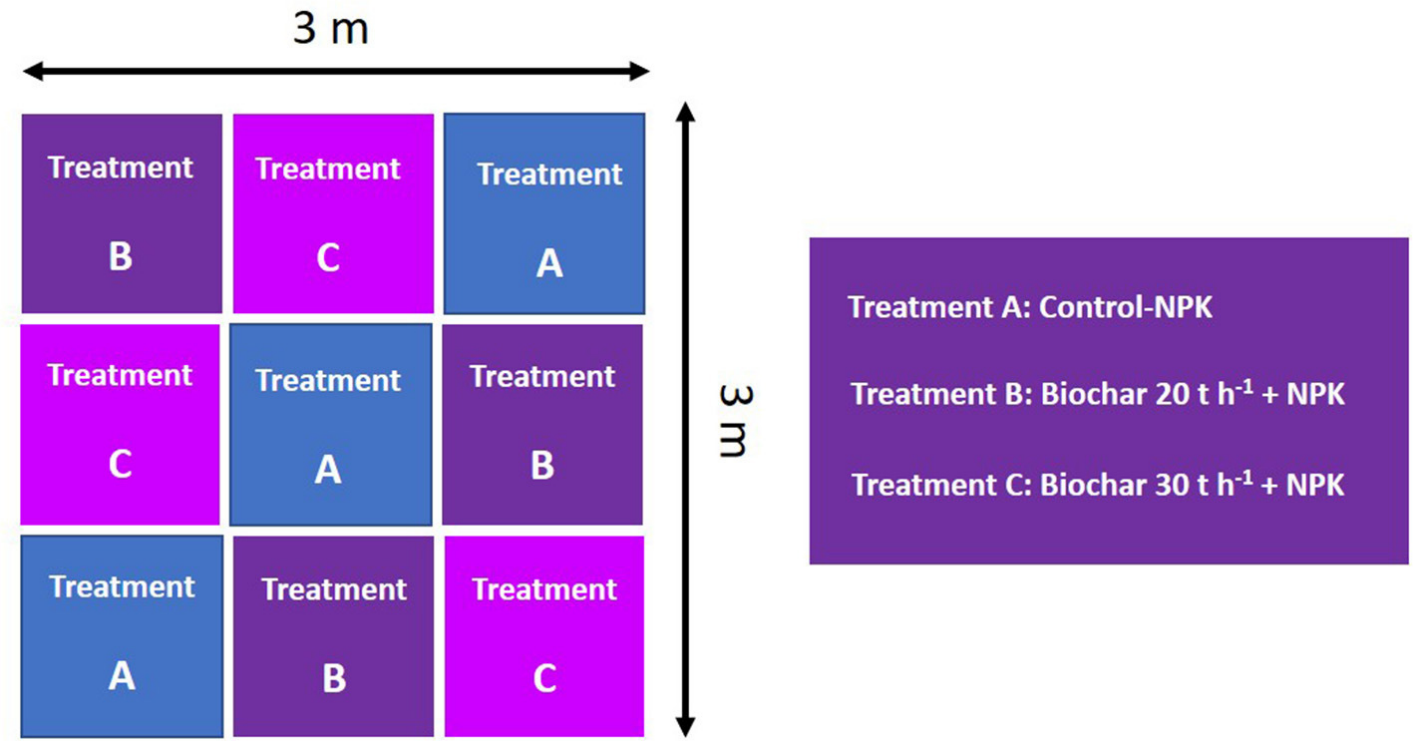
Field experiment design with three modalities (three replicates of each). Each individual plot had an area of 1 m^2^. Treatments are as follows: Treatment A is the control receiving NPK fertilizer N: P_2_O_5_:K_2_O at a dose of 40-40-50 tons ha^−1^, respectively; Treatment B, received 20 tons ha^−1^ of biochar in addition to NPK fertilizer as mentioned above and Treatment C received 30 tons ha^−1^ of biochar in addition to NPK fertilizer.

The study was carried out in nine plots, with an area of 1 m^2^ each in the year 2015. Three replicates of randomly arranged blocks received three types of treatment. In treatment A, three plots received only NPK fertilizer (control, commercially available NPK fertilizer, i.e., N:P_2_O_5_:K_2_O, 40-40-50 kg ha^−1^, respectively, recommended doses were calculated manually), another three plots under treatment B received the same dose of NPK and 20 tons ha^−1^ of biochar (5% by weight), and in treatment C, three random plots received the same dose of NPK along with 30 tons ha^−1^ of biochar (10% by weight), as explained in Figure 1. Biochar was added to the top 15 cm of soil and was mixed thoroughly, and soils were irrigated with saline water containing various ions (Na^+^, Ca^2+^/Mg^2+^, K^+^, HCO^3−^, and Cl in a concentration of 28, 10, 0.79, 1.2, and 35.6 meq L^−1^, respectively) via drip irrigation system (Gill et al., 2020). Physicochemical properties, such as electrical conductivity, soluble magnesium, calcium, chloride, potassium, and sodium content, were monitored constantly.

### 2.2. Soil sampling

Soil samples from unamended control and biochar-treated soil were collected at a depth of 0–15 cm from the Quinoa rhizosphere using a soil auger (16 cm in length). Samples were collected in sterile polybags and were transferred to the laboratory at 4°C for further processing. Three samples from each plot were collected and mixed properly to make a composite sample. Soil samples were collected in March 2017 from the shade house trial research station at International Center for Biosaline Agriculture (ICBA), Dubai, United Arab Emirates (25°05′49“ N and 55°23′ 25” E).

### 2.3. Determination of soil physicochemical properties

The soils were tested for various physicochemical properties, including electrical conductivity, soluble magnesium, calcium, chloride, potassium, and sodium content. The concentration of soluble magnesium and calcium was determined using standard titration with ethylene diamine tetraacetate (Lewis and Melnick, 1960). The soluble chloride in the soil was analyzed using a chloride analyzer (Sherwood Chloride analyzer 926, Cambridge, UK). The potassium and sodium concentrations in diluted soil samples were determined using a flame photometer (Jenway PFP7, UK) (Banerjee and Prasad, 2020). The available phosphorus in soil was determined using the standard colorimetric method of Deniges (Yuen and Pollard, 1955). The total nitrogen in the soil was estimated using the standard method of Kjeldahl (1883).

### 2.4. DNA extraction and illumina sequencing

Genomic DNA was prepared from a total of nine composite soil samples and collected from the three fields for each treatment. Power Soil DNA Extraction Kit (MO Bio Laboratories, Inc., Solana Beach, CA) was used to extract the DNA from 10 g of homogenized soil, according to the manufacturer's protocol. The purity and concentration of DNA were checked using a Qubit fluorometer (Thermo Fisher Scientific, USA). The extracted DNA was stored at −20°C until further use.

The diversity of bacterial, archaeal, and fungal communities in the samples was determined by amplifying the V3-V4 regions of bacterial and archaeal 16S ribosomal RNA (rRNA) genes and the ITS1 gene of fungi. The 16S rRNA gene amplicons were generated from 10 ng of the extracted DNA samples using a set of universal primers for the 16S rRNA gene and the ITS gene. These amplicons were checked on 1% agarose gel. To generate a V3-V4 amplicon (~460 bp), the PCR products as obtained above and a set of V3-V4 forward and reverse primers were used as described in our previous study (Khan et al., 2021). The non-specific fragments were removed using an AMPure XP beads kit (Beckman Coulter, CA, USA, Cat# A63882). The ITS fragments for fungi were also amplified using the same protocol, except for different PCR cycles and primers. For the library preparation, NEBNext Ultra DNA Library Prep Kit for Illumina (New England Biolabs, UK, Cat# E7370L) was used. Unique barcodes were incorporated for each sample during library preparation. The fragment distribution in the prepared libraries was checked using D1000 Screen Tapes (Cat# 5067–5582, Agilent, CA, USA) and reagents (Cat# 5067–5583, Agilent, CA, USA). The prepared libraries were, then, pooled and diluted to the final optimal loading concentration before cluster amplification on the Illumina flow cell. For amplicon sequencing to generate paired-end reads, the Illumina HiSeq2500 instrument (Illumina, Inc., San Diego, USA) was used.

### 2.5. Bioinformatics and statistical analyses

The sequencing reads generated by HiSeq 2500 instrument was subjected to standard bioinformatics analysis. FASTQC was used to check the quality of the raw sequence using the base quality, base composition, and GC content. Fastq-mcf was used to trim the sequences, excluding low-quality reads (PHRED Score) for retaining high-quality reads for further analysis. Reverse and forward sequences were merged into paired-end reads. Spacer and conserved regions were removed from high-quality PE reads. USEARCH was used for the dereplication of the sequences using 97% sequence similarity values. UCHIME utility of USEARCH was used to remove chimeras (Edgar et al., 2011). OTUs were picked up for further analysis by using the RDP classifier and the green gene database. OTUs, thus, determined were aggregated at the genus level for downstream processing.

The raw sequences have been deposited in NCBI (Bethesda MD, 20894 USA) under Bio-Project accession number PRJNA888571, with 18 biosamples of 16S and ITS gene-based analysis. The OTUs obtained were analyzed using various R packages, including phyloseq, microeco, and vegan (Oksanen et al., 2012; McMurdie and Holmes, 2013). The alpha diversity in the samples was calculated using Shannon and Chao1 diversity indices using the microeco package. The plots were prepared using ggplot2. Venn diagrams showing the genera shared by different samples based on the tables of shared OTUs were also prepared using Calypso. Heatmap-based relatedness between samples was also calculated using the Calypso or phyloseq packages as described earlier (Khan and Khan, 2020). The correlation between microbial diversity and environmental parameters was also calculated using the vegan package. To infer whether the tested data were normally distributed, a *p*-value of <0.05 for calculating the Shannon index was used. Differentially abundant archaeal, bacterial, and fungal OTUs were analyzed using the vegan package.

The statistical analysis of physicochemical parameters was performed using Statistical Product and Service Solution (SPSS, version 28.0.0 software, IBM SPSS Statistics, USA). A comparison of physicochemical parameters between biochar amended and unamended samples was performed using one-way analysis of variance (ANOVA), followed by *post hoc* analysis with Tukey's HSD test. The level of statistical significance was set at *p*-values of <0.05.

## 3. Results

### 3.1. Influence of biochar treatment on soil physicochemical properties

The physicochemical properties of the biochar-treated and unamended control soil were tested. The results presented in Table 1 show marked improvement in chemical and physical soil properties, following treatment with biochar. The addition of biochar led to an increase in the concentration of soil organic matter (OM), and this increase was found to be concentration-dependent. OM increased by 32 and 7.9% in B (biochar 20 tons ha^−1^) and C samples (biochar 30 tons ha^−1^), respectively, compared to control or sample A. However, the total carbon (TC) decreased slightly with increasing doses of biochar concentration. Compared to the control, the water retention capacity of biochar-treated soil samples B and C improved significantly by 9.5 and 12.76%, respectively, supported by *p*-values of 0.005 and 0.0001, respectively. The pH of sample C increased slightly (4.5%) compared to the control. Electrical conductivity (EC) significantly increased in both B and C samples by 8.77 and 82.35%, respectively, compared to control soil (*p*-value = 0.00152). The biochar treatment also improved the mineral (Mg, Ca, Cl, K, Na, and P) status of the soil. In biochar-treated soil, total nitrogen (TN) levels increased significantly, in sample B, slightly higher nitrogen (11.99%) was detected compared to sample C (11.39%). When the changes with different doses of biochar were compared, it was found that 30 tons ha^−1^ (sample C) of biochar show a better impact than the lower dose of 20 tons ha^−1^ (sample B).

**Table 1:** Change in soil physicochemical properties treated with biochar.

**Parameters**	**Control + NPK**	**Biochar 20 t ha-1**		**Biochar 30 t ha-1**	
	**Avg** ±**SD**	**Avg** ±**SD**	***p-value***	**Avg** ±**SD**	***p-value***
Organic matter (%)	1.26 ± 0.061	1.66 ± 0.065	<0.0001	1.36 ± 1.447	<0.0001
Total carbon (%)	1.11± 0.05	1.05 ± 0.073	0.058^*^	0.85 ± 0.085	<0.0001
Water retention (%)	5.17 ± 0.19	5.66 ± 0.42	0.009	5.83 ± 0.33	0.0006
pH	7.30 ± 0.02	7.25 ± 0.08	0.098	7.63 ± 0.12	<0.0001
Electrical conductivity (dS/m)	4.25± 0.01	4.62 ± 0.14	<0.0001	7.75 ± 0.08	<0.0001
Mg (meq L-1)	5.93 ± 0.04	5.95 ± 0.13	0.623^*^	8.75 ± 0.08	<0.0001
Cl (mmol L-1)	29.07 ± 0.45	31.3 ± 1.23	0.0002	61.18 ± 1.09	< 0.00001
K (meq L-1)	0.45 ± 0.09	0.81± 1.46	<0.0001	1.3 ± 0.04	< 0.00001
Na (meq L-1)	27.53 ± 0.73	38.21 ± 1.47	<0.0001	48.93 ± 0.52	<0.0001
P (mg Kg-1)	34.64 ± 1.05	39.08 ± 0.35	<0.0001	44.93 ± 0.60	<0.0001
Total *N* (mg Kg-1)	230.57 ± 0.94	258.23 ± 1.28	<0.0001	256.85 ± 2.34	<0.0001

Significant changes (p-value of <0.05) in biochar-amended soil compared with control by one-way ANOVA are shown. Values marked with asterisks are not significant.

### 3.2. Change in microbial community structure in response to biochar treatment

The sequences obtained had an average read length of ~300 base pairs, and a total of 178,835 high-quality reads were used for the analysis. The sequences were assigned to OTUs based on 97% sequence similarities. A total of 16 OTUs of archaea, 1,979 OTUs of bacteria, and 24 OTUs of fungi were observed in the samples.

**Figure 2:**
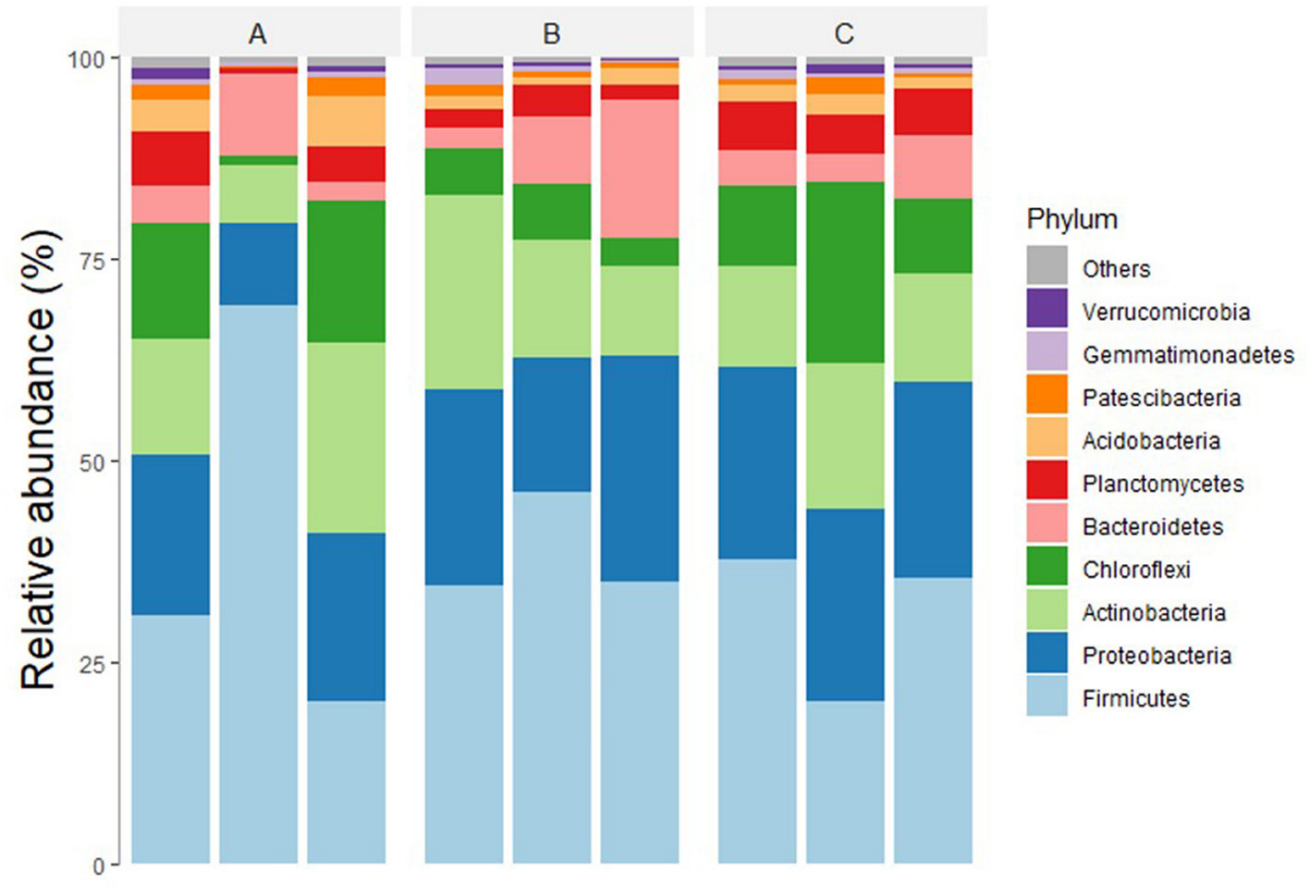
Relative abundance of major bacterial phyla in treatments **(A–C)**, receiving 0, 20, and 30 tons ha^−1^ of biochar with NPK. Microbial communities in all three treatments were analyzed in triplicates.

In the taxonomic rank analysis of the OTUs, the bacteria were found to be members of 33 phyla, 122 classes, 359 orders, 687 families, and 1,148 genera. The most abundant bacterial phyla in all the samples in descending order were Bacillota (synonym Firmicutes), Pseudomonadota (synonym Proteobacteria), Actinomycetota (synonym Actinobacteria), Chloroflexota (synonym Chloroflexi), Bacteroidota (synonym Bacteroidetes), and Planctomycetota (synonym Planctomycetes), accounting for 80–95% of all OTUs (Figure 2). Other phyla with significant populations were Verrucomicrobiota (synonym Verrucomicrobia), Acidobacteriota (synonym Acidobacteria), Nitrospirota (synonym Nitrospirae), and Gemmatimonadota (synonym Gemmatimonadetes).

**Figure 3:**
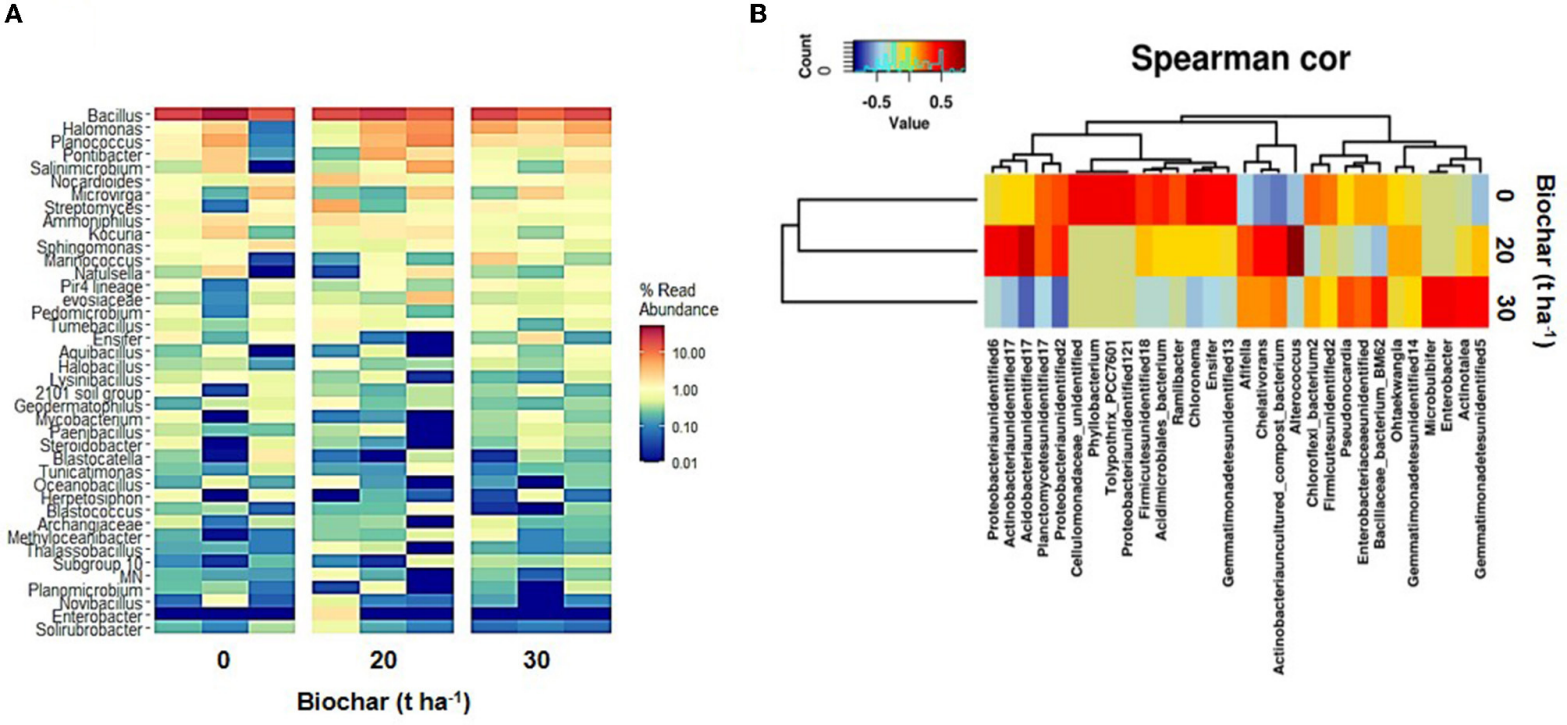
Heatmap showing 40 most predominant bacterial genera observed in soil samples treated with different doses of biochar **(A)**. **(B)** Shows a Spearman correlation heatmap based on the bacterial community and various doses of biochar.

**Figure 4:**
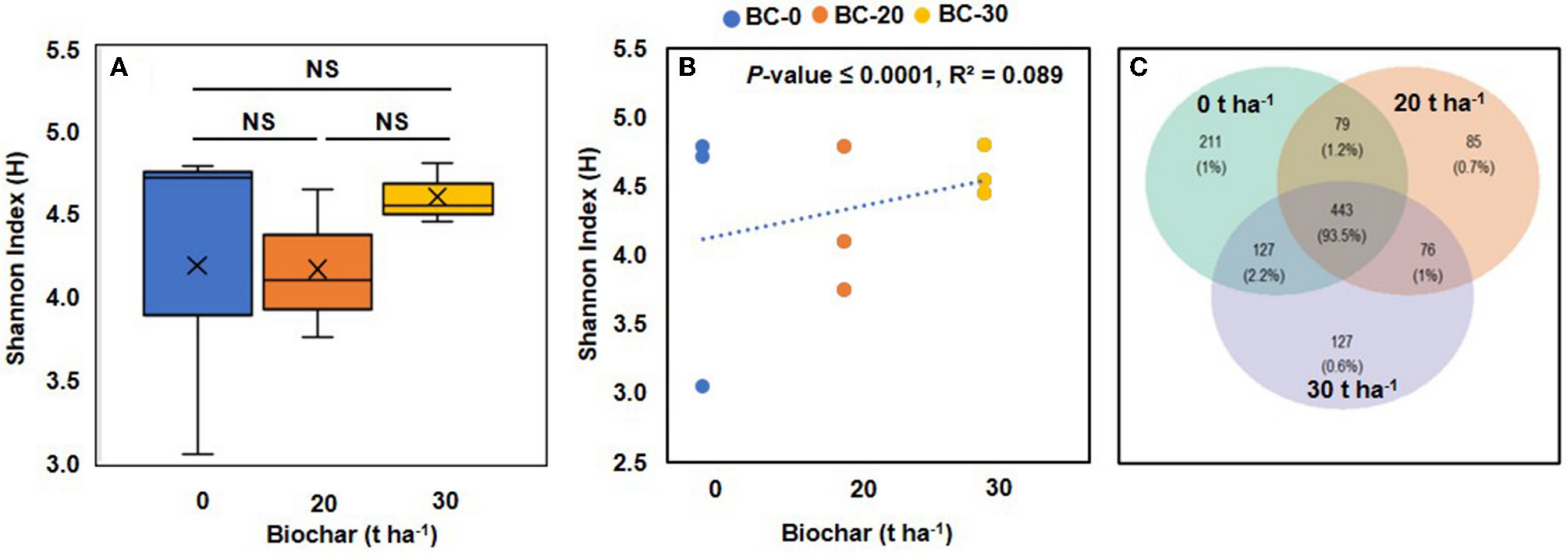
Boxplots illustrating alpha diversity (Shannon index) in bacterial microbiomes of treatments with different concentrations of biochar **(A)**. **(B)** Shows a correlation between the Shannon diversity index and increasing dose of Biochar. A positive correlation between bacterial diversity and increasing concentration of biochar was observed supported by a significant *p*-value. **(C)** Venn diagram showing the numbers of bacterial genera shared by treatments with different biochar concentrations. NS, not significant.

The analysis at the genus level shows that the 10 most abundant genera in the samples were *Bacillus, Halomonas, Planococcus, Pontibacter, Salinimicrobium, Nocardioides, Microvirga, Streptomyces, Ammoniphilus*, and *Kocuria*. The abundant bacteria of marine origin observed in this study were *Planococcus, Halomonas, Salinimicrobium, Marinicoccus*, and *Halobacillus*. Figure 3A shows a heatmap of 40 abundant bacteria, and Figure 3B and Supplementary Figure 1 show the Spearman correlation between various treatments and bacterial community diversity. The Spearman correlation shows that biochar-treated samples (samples B and C) group with each other more closely than the control sample (sample A). The Venn diagram depicting the shared genera between the samples clearly shows that the maximum number of genera is shared between the control and sample C (Figure 4C). The analysis of differentially abundant genera suggests that in biochar-treated samples, a few genera with known plant growth-promoting activities were differentially present (Supplementary Figure 2). Four bacterial genera that are enriched in biochar-treated soil include well-known nitrogen fixer *Mesorhizobium*, a PGPR biocontrol bacterium *Amycolatopsis* with activity against plant pathogen, marine sulfur-oxidizing bacterium *Sulfurifustis* with PGPR activity, and *Nitriliruptoria* with unknown function (Weir et al., 2004; Grayston and Germida, 2011; Gopalakrishnan et al., 2019).

The alpha diversity in the control (treatment A) and biochar-treated samples was calculated in terms of the Shannon index using the vegan package. The values of the Shannon index varied considerably in control samples but were more stable in biochar-treated samples with a shift toward a higher range in samples B and C (Figure 4A). When a correlation between the Shannon index and doses of biochar was plotted, it showed a positive correlation which was supported by a *p*-value of 0.0000039 (Figure 4B).

**Figure 5:**
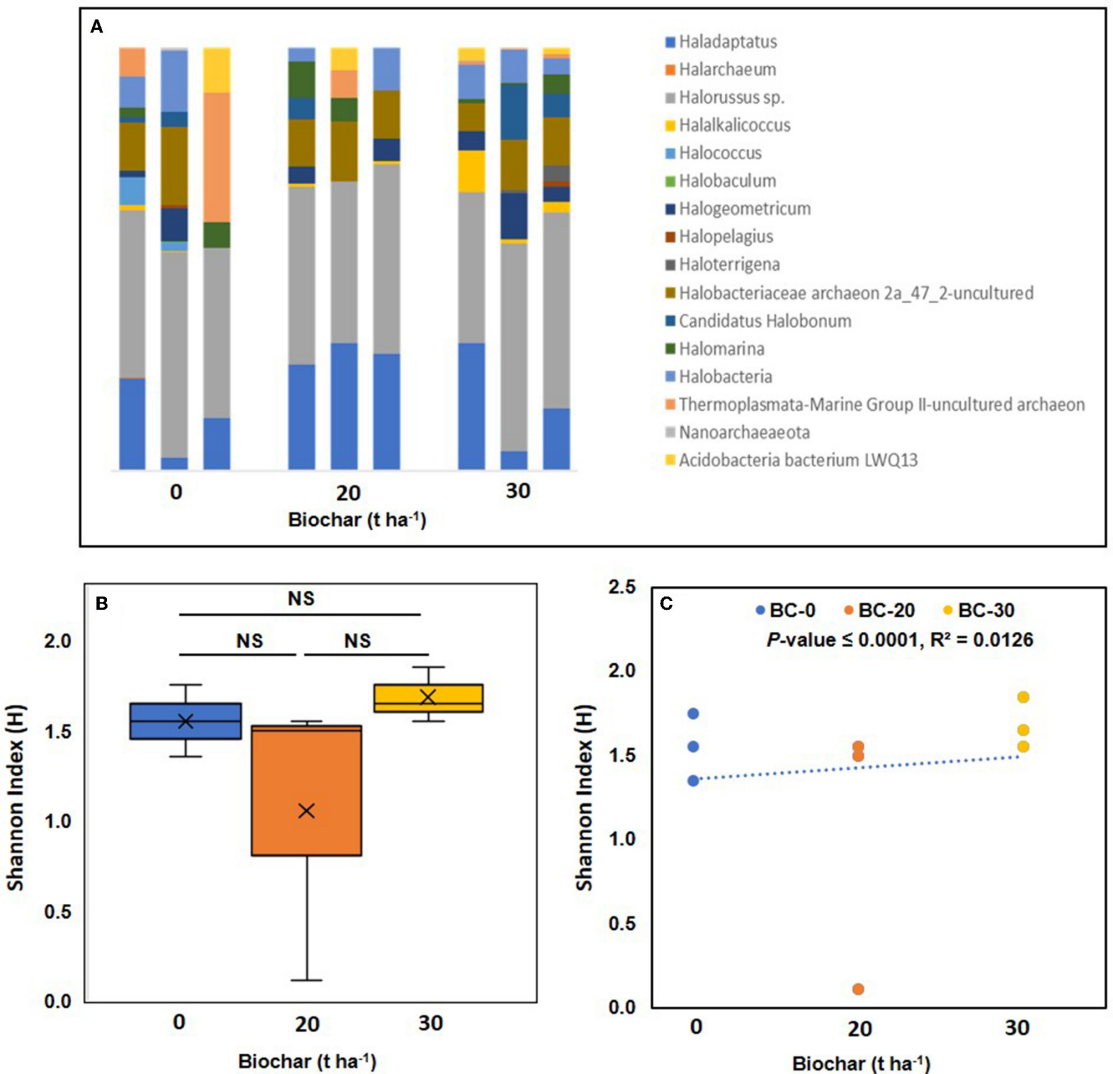
Relative abundance of archaeal genera in the biochar-treated and untreated soil samples in triplicates. **(A)** Shows the relative abundance of different archaea genera in the three treatments. **(B)** Shows a box plot of the alpha diversity of the archaea communities in the three treatments. Where means of observed Shannon index their median (lines in the boxes), and lowest and highest values are plotted. **(C)** Shows a correlation between the Shannon diversity index and increasing dose of Biochar. A positive correlation between bacterial diversity and increasing concentration of biochar was observed and supported by a significant *p*-value. NS, not significant.

Compared to bacterial communities, very few archaea were detected in the samples. In total, 16 archaeal genera were detected in the library of nine samples; the most abundant archaeal phylum was Euryarchaeota, followed by smaller populations of Nanoarchaeota and Thaumarchaeota. The relative abundance of major archaea genera is shown in Figure 5A. The five most abundant archaeal genera found in all samples were *Halorussus*, followed by *Haladaptatus* and *Halococcus*. The box plot of the Shannon index shows the highest diversity in sample C, while in sample B, the Shannon index varied considerably from sample to sample, and values of the Shannon index in the same range were also observed in the control sample (Figure 5B). When the Shannon index was plotted against the dose of biochar, a positive correlation with a *p*-value of 0.0035 was observed (Figure 5C).

**Figure 6:**
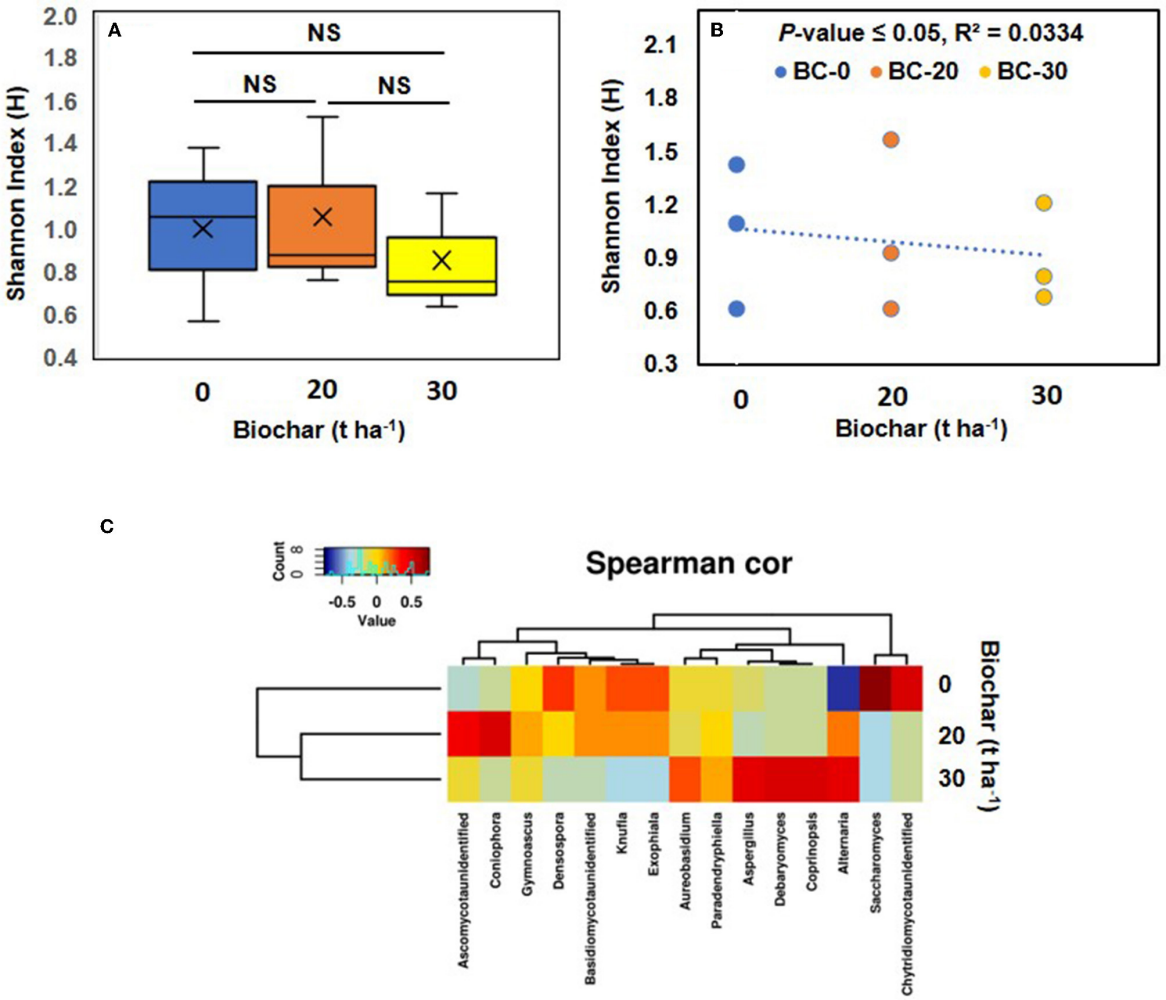
Fungal diversity. **(A)** Shows the alpha diversity of the fungal community in the three treatments. Where means of observed Shannon index their median (lines in the boxes), and lowest and highest values are plotted. **(B)** Shows a correlation between the Shannon diversity index and increasing dose of Biochar. A negative correlation between fungal diversity and increasing concentration of biochar was observed and supported by a significant *p*-value. **(C)** Shows a Spearman correlation (Spearman cor) heatmap based on the fungal community and various doses of biochar. The clustering of samples treated with biochar resulted in a change in the fungal community compared with the control. NS, not significant.

When the fungal genera were analyzed, the least diverse population was found in sample C, and the most diverse fungal population was observed in control sample A, showing a shift in the fungal community following biochar amendment. When the Shannon index was plotted against the concentration of biochar, it was found that the values within various treatments varied considerably (Figure 6A). When a correlation between the Shannon index of various treatments was plotted against the biochar concentration, a negative correlation was observed (Figure 6B). A Spearman correlation shows that the biochar-treated sample group together and separately from the control, showing a shift in the fungal community, following amendment with biochar (Figure 6C). Interestingly, some of the fungi detected are known to benefit plant partners, such as *Aspergillus*, which is known to produce plant growth hormones (Hung and Lee Rutgers, 2016). Although the increase in the population of *Aspergillus* was not significant with a *p*-value of 0.38, the population increased slightly in sample C.

### 3.3. Correlation of soil physicochemical properties with the Shannon index

**Figure 7:**
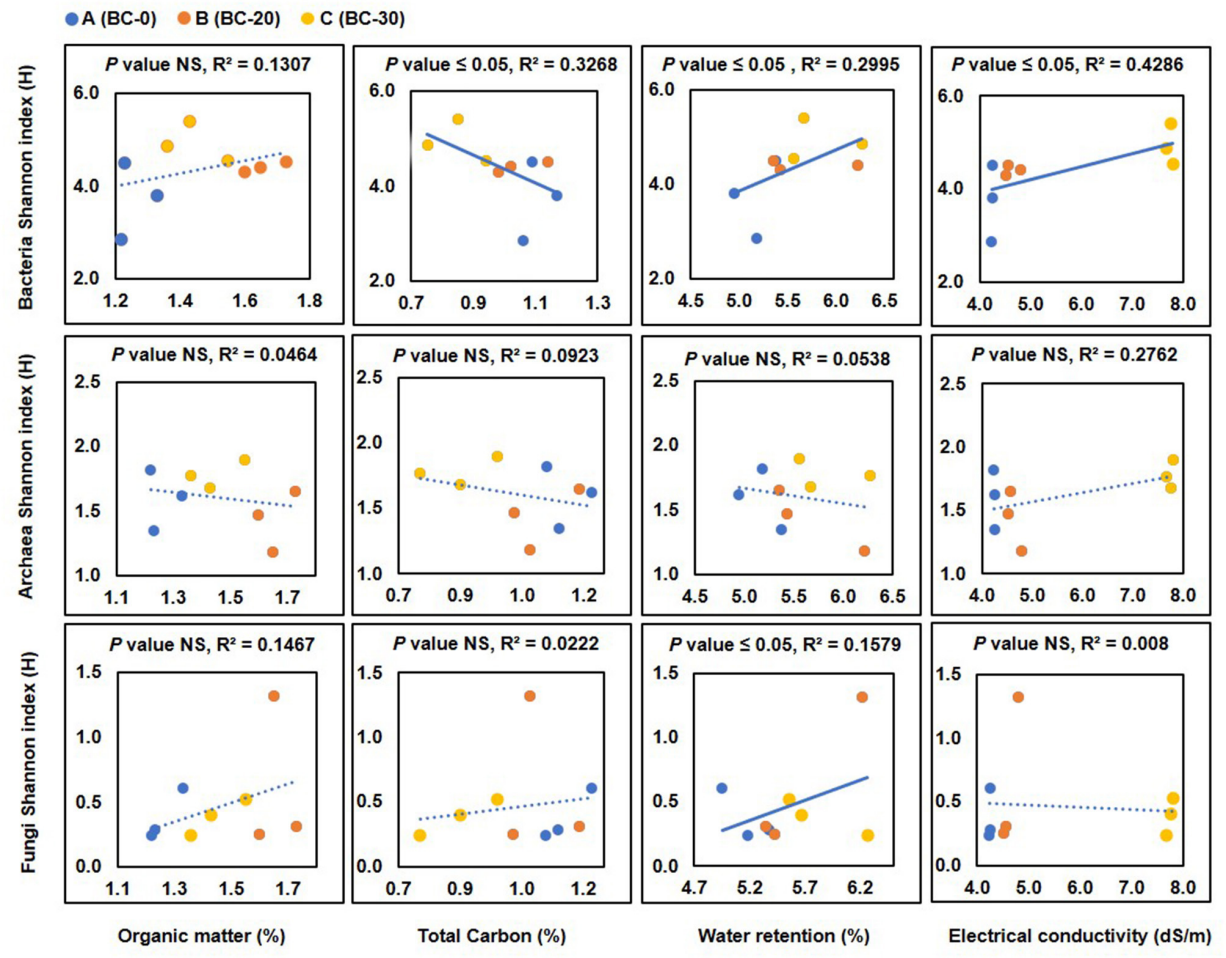
Correlation of bacterial, archaeal, and fungal diversity in terms of Shannon index with changes in organic matter (OM), total carbon (TC), water retention (WR), and electrical conductivity (EC) following treatment with various concentrations of biochar, **(A)** control, **(B)** 20 t ha^−1^ and **(C)** 30 t ha^−1^. Values of the parameters are plotted on the x-axis and values of the Shannon index are plotted on the y-axis. NS, not significant. Significant values are marked with a solid line, while insignificant values are shown as dotted lines.

Soil amendment with biochar altered the soil's physicochemical properties, which consequently influenced the soil microbial community. This change in physicochemical properties was plotted against the microbial diversity in terms of the Shannon index (H'). When the bacterial diversity (Shannon Index) was plotted against the organic matter content, a slightly negative correlation between an increase in the organic matter content of soil and diversity in terms of Shannon index values was observed. The total carbon in the soil decreased slightly with increasing doses of biochar, and the values of the Shannon index also increased, indicating a significant (*p*-value = 0.0024) increase in biodiversity (Figure 7A). EC increased with the increasing concentration of biochar (*p*-value = 0.00152). The values of the Shannon index increased with increasing values of the electrical conductivity. When the diversity of archaea in terms of the Shannon index was plotted against these physicochemical properties, no significant correlation was observed (Figure 7B). Similarly, in fungal diversity analysis also, no direct correlation between physicochemical properties and diversity was observed (Figure 7C). It is to be pointed out here that only a few taxa for both archaea and fungi were detected, and this lack of extensive data may have led to the lack of any significant correlation.

## 4. Discussion

Soil amendment with biochar is becoming increasingly popular as it improves and sustains soil fertility. Biochar is one of the widely used soil conditioners which, in addition to improving soil fertility, can also be used for carbon sequestration (Woolf et al., 2010; Lehmann et al., 2011). Biochar improves soil fertility through the improvement of soil physicochemical properties such as pH, water retention capacity, organic matter content, and soil microbial community structure (Sohi et al., 2009; Zhang et al., 2018). Most of the studies have been conducted in pots or for shorter time periods. In this study, we report the influence of biochar amendment on the fields with low-carbon arid soils irrigated with saline water.

The change in physicochemical properties was checked after 1-year of the biochar treatment, and it was observed that the biochar treatment improved the organic matter content, EC, water retention, and soil nitrogen, improving the overall nutrient content of the soil. However, the total carbon decreased slightly. It has been demonstrated by other authors that the change in the microbial community with the addition of pyrogenic organic matter results in carbon loss from the soil, especially in low-carbon soil (Whitman et al., 2021). The nutritive and structural properties of biochar play an important role in changing the microbial community structure and activity in the soil (Zhang et al., 2018). In saline–alkali soils, EC is an important driving force for change in the microbial community (Wang et al., 2019).

It has been demonstrated that biochar significantly increases the fungi-to-bacteria (F/B) ratio and the gram-positive to gram-negative (G+/G-) ratio (Zhang et al., 2018). Moreover, this increase was quite significant in dry lands. Contrarily, a decrease in the bacteria-to-fungi ratio and bacteria-to-archaea ratio was observed in our study, which was conducted in low-carbon sandy soil. It is argued that the addition of organic matter to soil may not always translate into an increase in microbial diversity or number as different microbial communities respond differently to added organic matter. This may be due to the difference in the carbon use efficiency, carbon assimilation rates, and the ratio of various microbes in the soil (Whitman et al., 2021). Furthermore, the type of biochar also influences the microbial community structure.

Microorganisms offer a multitude of plant growth-promoting benefits to plants, and some groups of microorganisms are so crucial for plant growth that even plants are known to recruit these microbial populations with PGPR traits through the production of specific root exudates (Khan, 2022). This study focused on understanding the change in the microbial community in response to amendment with biochar with special reference to the microbial community shift toward plant growth promotion. It is also important to understand whether the shift in microbial community structure is derived from the change in physicochemical properties of the soil caused by biochar amendment. Change in archaeal, bacterial, and fungal microbial communities was studied. The analysis of fungal, bacterial, and archaea population shows that the addition of biochar helps in retaining the soil microbial community diversity, and significant changes in their diversity were observed, following 1-year treatment with biochar. On the contrary, some earlier studies report little or almost no change in microbial diversity, following biochar treatment (Anderson et al., 2014). In this study, when the Shannon index values were plotted with the increasing dose of biochar, the archaea diversity was highest in fields treated with 30 tons ha^−1^ of biochar, and a relation supported by a *p*-value of 0.0035 was found. Similarly, for bacteria, also an increase in bacterial diversity with increasing concentration of biochar was observed. This was also supported by a *p*-value of 0.0000395. However, a decrease in fungal diversity with an increasing concentration of biochar was observed and supported by a *p*-value of 0.025. Various reports on the response of the microbial community to biochar addition are available. In one such study, no change in archaea, an increase in bacterial diversity, and a very significant change in fungal diversity were observed as a consequence of biochar treatment (Abujabhah et al., 2016). Other studies report that fungal diversity changes remarkably following treatment with biochar (Shasha, 2017). These variations in observations may be due to various reasons such as quality of biochar, source of biochar, type of soil, type of microbial community, climatic condition, and water quality (Agegnehu et al., 2017).

Our previous study on Saudi soil and wastewater of the gulf indicates some important facts (Khan and Khan, 2020; Khan et al., 2021). One is the presence of a significant population of marine bacteria, which may be due to the widespread use of desalinated seawater in this region. Second, the presence of thermophilic bacteria due to the prevailing climatic condition and the presence of bacteria that can utilize very simple compounds as nutrients indicate the lack of nutrients in arid soil. In this study, *Bacillus* was detected as one of the most dominant genera may be due to its ability to survive under harsh environmental conditions through spore formation (Masood et al., 2022). Another dominant genus is *Microvirga*, which has been reported as an endosymbiont of the specific legume plant *Lupinus micranthus* found in the arid region and is known to fix nitrogen (Radl et al., 2014; Msaddak et al., 2017). Interestingly, the shift in microbial community that took place as observed in this study shows enrichment of a few plant growth-promoting microbes, including some genera of bacteria, fungi, and archaea especially with a dose of 30 tons ha^−1^. An increase in the population of N_2_-fixer, sulfur oxidizer, or bacteria-exhibiting activity against plant pathogens was observed. These include *Mesorhizobium, Amycolatopsis, Sulfurifustis*, and *Nitriliruptoria*. Previous studies have documented the rise in the population of such bacteria, following the treatment with biochar (Schmalenberger and Fox, 2016). Even many of the archaeal genera adapted to high salt concentration with PGPR traits were detected after the treatment with biochar. Members of genera, such as *Halococcus* and *Haloterrigena*, are known to exhibit PGPR activities such as phosphate solubilization and hydrolysis of organic macromolecules (Yadav et al., 2015; Ding et al., 2020). Isolation of such microbes exhibiting PGPR activities, thermotolerance, and salt tolerance can help in designing a consortium-based biofertilizer for improving arid soil fertility irrigated with seawater.

## 5. Conclusion

The treatment of the low-carbon arid soil with indigenously prepared biochar improved soil physicochemical properties and its nutritional status. The microbial diversity including the bacteria and archaea changed significantly compared to unamended soil. Fungal diversity decreased with the biochar treatment. The change in microbial community was characterized by the presence of a few PGP microbes as suggested by the analysis of differentially abundant genera. The change in physicochemical properties due to the biochar treatment was positively correlated with the increase in the diversity of bacteria and archaea. This study, thus, concludes that the biochar treatment helped in sustaining soil fertility through the sustenance of soil microbial diversity. A typical characteristic of the soil microbial community was the presence of thermotolerant and salt-tolerant microbes which may be due to the climatic condition and the use of saline water for irrigation.

## Data availability statement

The datasets presented in this study can be found in online repositories. The names of the repository/repositories and accession number(s) can be found below: https://www.ncbi.nlm.nih.gov/genbank/, PRJNA888571.

## Author contributions

MK and SK: formal analysis, investigation, and writing—original draft. AS: methodology. MK: project administration. All authors contributed to the article and approved the submitted version.
