# Supplementary figures and images for: Indigenously produced biochar retains fertility in sandy soil through unique microbial diversity sustenance: a step toward the circular economy
Source: Front Microbiol. 2023 Jun 27;14:1158784. doi: 10.3389/fmicb.2023.1158784 (PMC10335804; doi:10.3389/fmicb.2023.1158784)

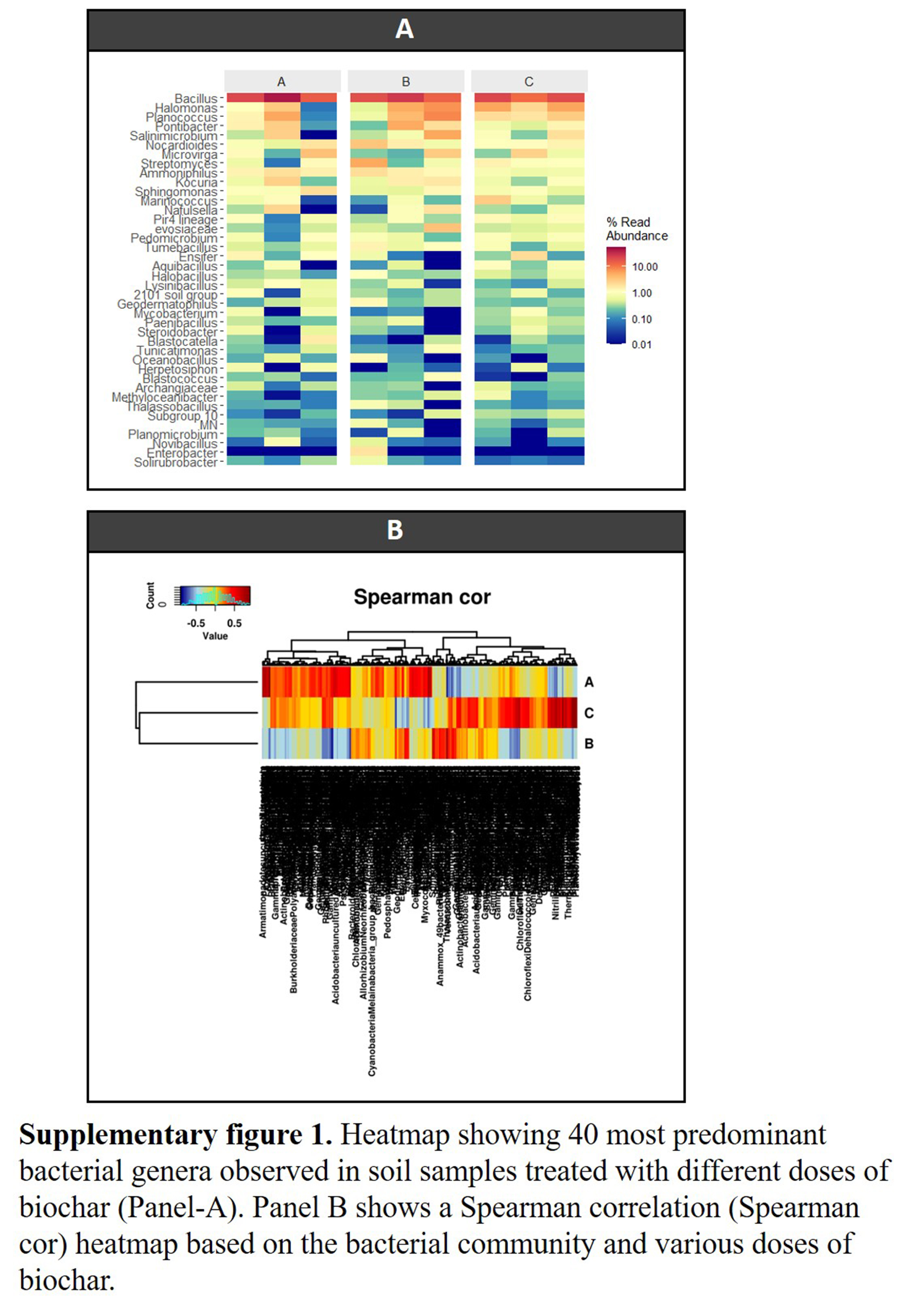

Supplement: Supplementary file 1 [file Image_1.JPEG]

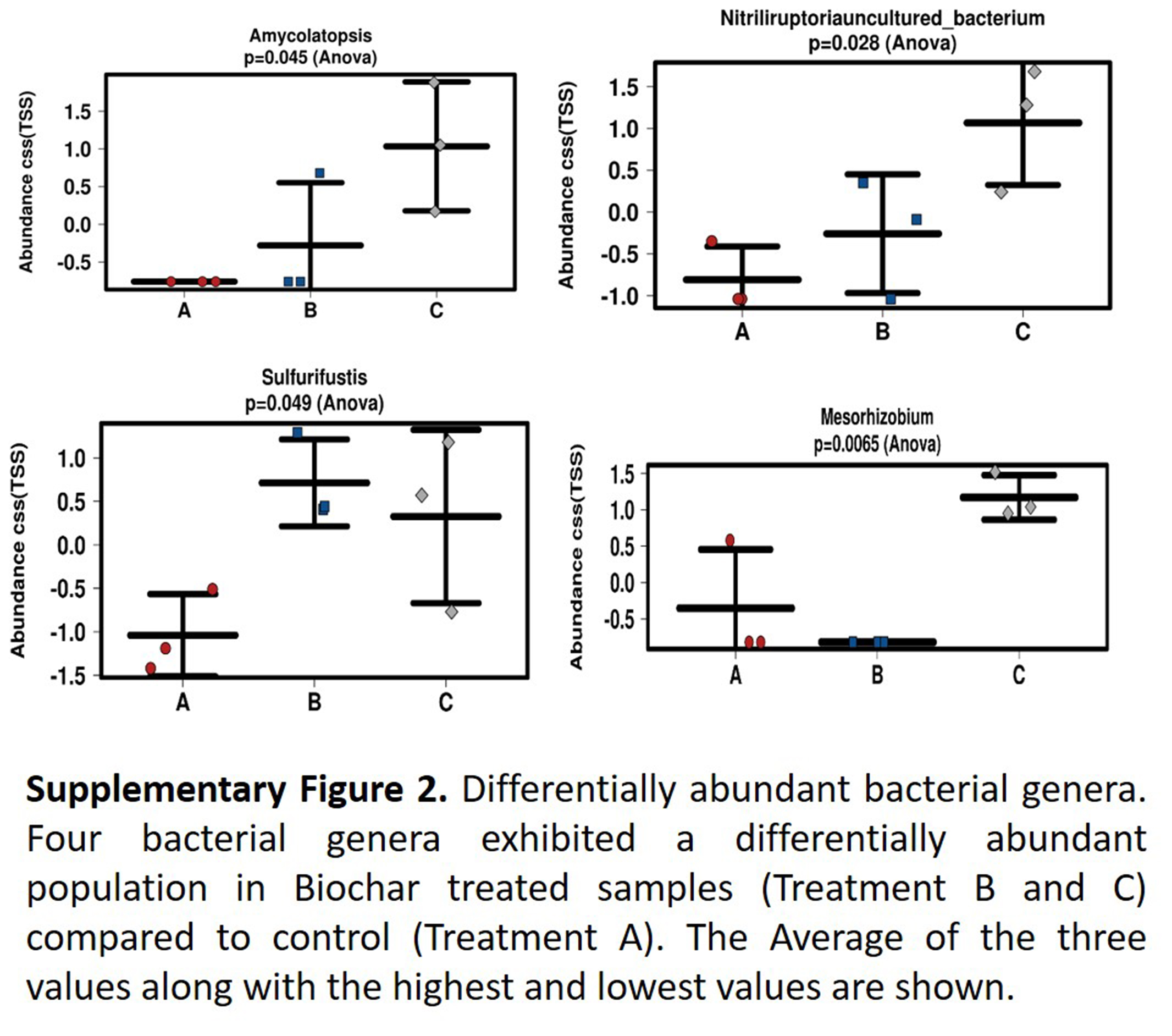

Supplement: Supplementary file 2 [file Image_2.JPEG]
